# Towards a preventive strategy for complaints of arm, neck and/or shoulder (CANS): the role of help seeking behaviour

**DOI:** 10.1186/s12889-016-3853-8

**Published:** 2016-11-28

**Authors:** Vivian E. J. Bruls, Nicole W. H. Jansen, Rob A. de Bie, Caroline H. G. Bastiaenen, IJmert Kant

**Affiliations:** Department of Epidemiology, Care And Public Health Research Institute, Maastricht University, Universiteitssingel 40, Maastricht, MD 6200 The Netherlands

**Keywords:** Complaints Arm Neck Shoulder, Help seeking, Preventive strategy, Employees, Students

## Abstract

**Background:**

When developing an effective early preventive strategy for employees and students with CANS (Complaints of Arm, Neck or Shoulder, not caused by acute trauma or systemic disease), insight in help seeking behaviour and knowledge of factors associated with help seeking behaviour within the target population, is a prerequisite. The aim of this study was to examine whether perceived hindrance is associated with help seeking behaviour, specifically in employees and students identified with CANS. Additionally, the associations of factors related to functioning and participation, work-environment and demographics with help seeking behaviour were explored in these groups.

**Methods:**

A cross-sectional survey was conducted among employees and students of two universities in the South of the Netherlands. The questionnaire included questions to assess (1) demographics, work/study and activity related factors (2) experience of CANS (3) perceived hindrance (4) help seeking behaviour. A subpopulation of the survey, consisting of those employees and students with self-reported CANS, received additional questionnaires to examine the impact of (1) participant characteristics (2) complaint and health related variables (3) functioning and participation (4) work-environment and social support, on help seeking behaviour.

**Results:**

37.3% of the employees and 41.4% of the students reported CANS. Of these, respectively 43.3% and 45.5%, did not seek help *and* had no intention to seek help either. Employees and students who had not sought help reported less hindrance, less perceived disabilities and shorter duration of complaints, compared those who did seek help. Employees and students within this group who had also *no intention* to seek help, perceived fewer disabilities and reported shorter duration of complaints.

**Conclusion:**

The absence of help seeking behaviour in respondents with CANS is a bottleneck for implementation of preventive strategies. In employees and students with CANS, help seeking behaviour is primarily determined by factors related to experienced hindrance. Our findings emphasize the need to tailor preventive strategies, in order to optimize screening and participation in early interventions for CANS.

## Background

Complaints of arm, neck and/or shoulder, not caused by an acute trauma or systemic disease (CANS), are common in the Western world [1]. A study on the prevalence of CANS in the Dutch general population, described a 12-month prevalence of 37% and a point prevalence of 26% [2]. Besides personal suffering and distress, upper-extremity complaints have substantial economic and social impact. Although currently no prevalence rates of CANS, as defined by the CANS model [1], are available for the working and student population, upper-extremity disorders (UEDs) in general are found to be highly prevalent in the working and student population [3]. Often, upper-extremity complaints are initially localized in one anatomical area, but may evolve into more widespread symptoms [4]. The symptoms are usually mild at first, but may worsen gradually over time and can develop to a level at which they become chronic and disabling. These severe and disabling UEDs, represent a major cause for temporary and sometimes long-term sickness absence from occupational work or study [5–7]. Once on sick leave, individuals with musculoskeletal complaints may encounter difficulties to return to work or to resume study activities [8–11].

Until now, much attention has been paid to interventions aimed at individuals with severe and disabling symptoms, who are already absent from work. However, the effectiveness of treatments with the aim to improve the ability to return to work or to reduce the duration of sick leave once complaints have become chronic and disabling, is limited [12, 13]. Therefore, it is hypothesized that a better and more effective strategy would be to intervene in an earlier stage, before absenteeism and (work) disability actually occur. Previous studies revealed that early proactive treatment, when musculoskeletal complaints are beginning and are still mild in nature, lead to a significant decrease in pain intensity, improvement of activity levels and reduction of chronic problems [14, 15]. Therefore, a preventive approach for CANS should be considered aiming at individuals with beginning and mild symptoms, who are still at work. Regarding employees who are affected by CANS, a parallel can be drawn between this group and employees with mental health complaints. Also within the latter population, it was demonstrated that work resumption remains difficult once sickness absence occurred and reintegration has limited effects [16–19]. A previous study of Lexis et al. demonstrated that an early preventive approach aimed at employees who are at high risk of future sickness absence and who have a mild level of depressive complaints, proved effective in preventing future long-term sickness absence and major depression [20]. Notwithstanding this, in an additional study it was demonstrated that from those employees, identified with mild to severe depressive complaints, 43% of the respondents reported not to experience health complaints at that moment and had no intention to seek help [21]. This implies a point of concern, since the experience of health complaints is a prerequisite for help seeking behaviour. Given this, precisely those individuals with a mild level of complaints, at risk for future sickness absence and/or severe health complaints, are unlikely to display any help seeking behavior. As a result, the screening uptake of these individuals will be low, and they might refuse participation in an early preventive intervention or may not be adherent to a treatment once participation is initiated. To develop an effective early preventive strategy with optimal participation and treatment adherence, insight in the determinants of help seeking behaviour within the target population is fundamental [21]. A model which is applicable in explaining help seeking behaviour is the Health Belief Model (HBM) [22–24]. According to the HBM, help seeking behaviour is primarily determined by the subjective experience of health complaints, defined as ‘illness’ [25–28]. Furthermore, the model states that help seeking is determined by the perceived threat of the health complaints. If the individual perceives the health complaint as having serious consequences in terms of functioning and participation, help seeking behaviour may be induced [22]. In this context, it might be expected that the help seeking process may be different within the working and student population, when compared with the general population. Employees and students might be more alert to health complaints in an earlier stage of the disease compared to the general population, and might anticipate more limitations in functioning and participation as the illness may intertwine with work or study. The interference of illness with work or study, and the threat of sickness absence or study discontinuation, might be a reason for seeking help more promptly [28]. In turn, factors related to the work environment, such as job demands, social support and (physical) workload can influence the perceived threat of the disease in terms of functioning and participation and, hence, help seeking behaviour [22–24]. Apart from health and work related factors, also demographic factors such as age, gender, educational attainment, religion and culture, can be associated with the experience of health complaints and the help seeking process [22–24]. In previous studies, women were usually found to experience more health complaints [29, 30] and demonstrate different help seeking behaviour than men [31], both in the general as in the working population. Furthermore, employees identified with a low or medium educational attainment were found to experience more health complaints, compared to employees with a high educational attainment [21].

In order to develop an effective early preventive strategy for employees and students with CANS, insight in help seeking behaviour and knowledge of factors which are associated with help seeking behaviour within this target population, is a prerequisite. Since the initial symptoms of CANS are usually mild and restricted to one anatomical area, and do not immediately result in limitations in functioning or hindrance in participation, the initiative to seek help for these complaints is often postponed [32]. Mild symptoms of CANS are frequently seen as relatively harmless, self-curing and as ‘part of the job’, especially in positions with heavy physical workload or frequent computer tasks. Especially within a population with beginning musculoskeletal complaint as CANS, information on factors which influence the help seeking process will be of great value to optimize participation and the effect of an early preventive strategy. To our knowledge, so far no studies aimed at quantifying help seeking behaviour and examining determinants of help seeking behaviour, have been conducted among employees and students identified with CANS.

The objective of this study was to examine whether the perceived hindrance of the complaint is associated with help seeking behaviour specifically in employees and students identified with CANS and to explore the associations of factors related to functioning and participation, work-environment and demographics with help seeking behaviour in these groups. We used the HBM as a framework to examine the associations of different domains on help seeking behaviour.

## Methods

### Study populations and design

#### Study population and design to examine associations between perceived hindrance of CANS and help seeking behaviour

**Fig. 1:**
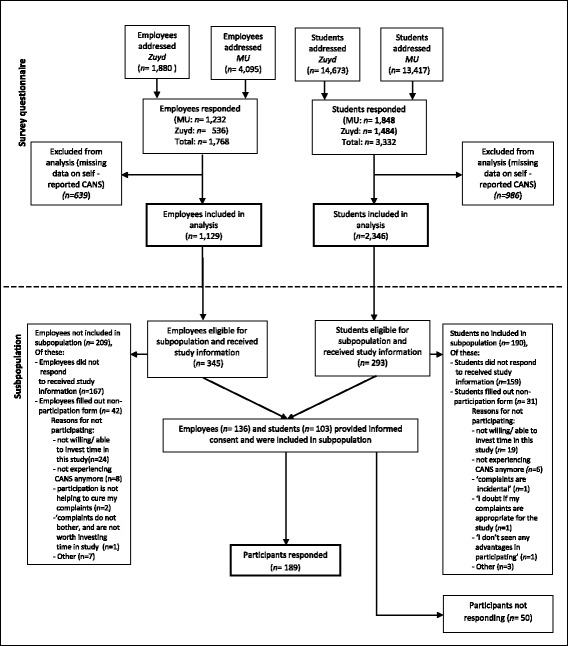
Study recruitment and response. Legend: MU, Maastricht University; Zuyd, Zuyd University of Applied Sciences

A cross-sectional survey was conducted among employees and students of two large universities in the Southern part of the Netherlands, Maastricht University and Zuyd University of Applied Sciences. From September 2013 through December 2013, 5,975 employees and 28,090 registered students were invited to participate in the survey (Fig. 1). The primary aim of the survey questionnaire was to gain insight in the prevalence of CANS within the university population in light of setting up a preventive strategy for CANS. Furthermore, data extracted from this survey questionnaire were analysed to examine whether an association could be established between perceived hindrance and help seeking behaviour within the population of employees and students with CANS. The survey questionnaire included questions on demographic and work and activity related variables, the experience and hindrance of CANS, complaint characteristics, and help seeking behaviour.

Employees and students were invited to participate in the survey by an invitation letter, sent by e-mail, which included a link for direct access to the digitalized survey questionnaire. Two weeks after the invitation letter, a reminder was sent to the non-respondents.

#### Study population and design to examine the impact of factors related to functioning and participation, work-environment and demographics

To examine the impact of factors related to functioning and participation, work-environment and demographics on the association between perceived severity of CANS and help seeking behaviour, a subpopulation consisting of employees and students with self-reported CANS is selected out of the respondents of the cross-sectional survey (Fig. 1). The survey included questions on selection criteria for the subpopulation. Eligible for inclusion in the subpopulation were employees or students with pain or complaints (defined as discomfort, numbness, skin discolouration, temperature differences, decreased function) in neck and upper extremities (shoulder, upper arm, elbow, forearm, wrist or hand) at this moment or within the past three months. Excluded were those with complaints caused by acute trauma (i.e., fracture or dislocation) or by any systemic disease, malignancy, prosthesis, amputation, congenital defect or a co-morbidity causing severe disability in daily activities. Employees and students who were pregnant were also excluded from participation in the subpopulation. When survey respondents fulfilled selection criteria an additional question was asked whether participants were interested in receiving study information to participate in the subpopulation. If respondents were interested, they received an email with attached study information. Respondents were requested to provide informed consent or fill out a non-participation form within two weeks. After two weeks a reminder was sent to non-respondents. If informed consent was provided, participants received an email with access to the online questionnaires within two weeks.

### Measurements

The *survey questionnaire* included questions to assess the self-reported information on:

1. Demographic characteristics, work/study and activity related factors

The following demographic factors were inventoried: Age, gender, and educational attainment. Educational attainment was defined as the highest level already completed (low = no education, primary school; medium = high school/ General Educational Development test or college; High = Bachelor’s degree, Master’s degree, advanced graduates work or PhD). The survey-questionnaire was tailored to the target population. The questionnaire intended for employees included questions on work-variables, whereas the survey questionnaire intended for students included study related questions. Employees were asked about employment status (part-time = ≤ 32 h/week, full-time= > 32 h/week), job category (support staff or scientific/teaching staff), years working in current job (<3 years versus ≥ 3 years), work absence due to CANS (no absence, < 2 weeks, 2 weeks-1 month, > 1 month), Visual Display Use (VDU) during working hours (scores were dichotomized into < 4 h/day and ≥ 4 h/day), additional paid job (yes/no). Students were asked about study load (part-time = ≤20 h/week, full-time > 20 h/week), additional job (yes/no), study related VDU use (<4 h/day, ≥ 4 h/day), study delay due to CANS (yes/no).

Activity related factors included: sports participation ‘Do you participate in sports for at least two times/week (performing during 30 min’ physical heavy exercise that makes you sweat)?’, playing a musical instrument (‘Does your job/ study involve playing one or more musical instrument(s)?’, playing a musical instrument in leisure time (yes/no), VDU use during leisure time (score were dichotomized into <2 h/day and ≥ 2 h/day).

(2) CANS

The experience of CANS was operationalized by the following questions: The first question was “Do you experience pain or complaints at neck, shoulder, upper arm, elbow, underarm, wrist, neck or fingers, at this moment or during the past three months?’ (yes/no). If this question was answered with an affirmative, additional questions were asked about severity or characteristics of the complaint: “For how long did you experience these complaints?” (scores were dichotomized in <1 month and ≥ 1 month); “Did you experience similar complaints before?” (yes, one time before; yes, more times before; no, never before) and a question on putative cause according to respondent: “What do you think is the cause of your complaints?” (work/study related, not work/study related). Furthermore, the respondent was asked whether complaints were caused by an acute trauma or systemic disease, malignancy, prosthesis, amputation, congenital defect or a co-morbidity causing severe disability in daily activities. Complaints at arm, neck or shoulder caused by an acute trauma, or systemic disease were *not* defined as CANS.

(3) Perceived hindrance of the complaints

The perceived hindrance of the complaints was assessed by the questions ‘Do the complaints lead to disabilities in your daily activities’ (yes/no) and ‘How often do the complaints hinder?’ (continuously, regularly, complaints do not hinder).

(4) Help seeking behaviour.

Questions on help seeking behaviour were modified from the questions that were applied in a study of Lexis et al. who investigated help seeking behaviour in employees with depressive complaints [21]. First, the person was asked: ‘Have you already sought help for these complaints of the arm, neck and/or shoulder?’. If the question was answered with an affirmative, the following question was: ‘By whom’? On the other hand, if the person indicated to have not sought help for the CANS already, the subsequent question was:’Do you have the intention to seek help for your pain/symptoms in the near future?’.

The *subpopulation*, consisting of employees and students with self-reported CANS, received *additional questionnaires* to examine the association of help seeking behaviour with:

1. Participant characteristics

Participants were questioned on gender, age, marital status (unmarried/never been married, divorced/widow, married/living together), and on the presence of children in the household (yes/no). Furthermore, participants were asked’Do you consider yourself Dutch/Non-Dutch?’.

(2) Complaints and health related variables

Complaints and health related variables included the following self-formulated items: ‘prognosis according to respondent’ (complaints will improve soon or are already improved, complaints will improve gradually and will finally be recovered, complaints will improve gradually but will never be fully recovered, complaints will stay the same or worsen), frequency of hindrance in past three months (complaints do not bother me, now and then recurrent, regularly recurrent, almost continuously), discomfort of complaints (no discomfort, now and then discomfort, regularly discomfort, almost continuously discomfort), and general health (moderately, good, very good, excellent). Furthermore, participants were asked if complaints worsen during work/study related activities (yes/no), if adaptations in work or study had taken place due to CANS (yes/no), if complaints diminish in leisure time (yes/no), if a specific diagnosis was established by a physician (yes/no), and on the onset of the complaints (gradually/ suddenly).

(3) Functioning and Participation

Functional limitations of the neck, shoulder, arm or hand were measured using a modified version Dutch and English version of the Disabilities of the Arm, Shoulder, Hand questionnaire (DASH) [33]. One item on sexual activities was excluded. The items on this 29-item questionnaire assess the ability to perform certain activities. These items were scored on a five-point Likert scale. Response scores were added up to a score ranging from 29 (no disability) to 145 (completely disabled). Higher scores represented more functional limitations.

To measure several aspects of individual’s participation and autonomy we used the Dutch and English version of the Impact on Participation and Autonomy Questionnaire (IPA) [34]. This questionnaire consists of the following five domains: autonomy indoors (7 items, domain score ranges from 0–28); family role (7 items, domain score ranges from 0–28); autonomy outdoors (5 items, domain score ranges from 0–20); social life and relationships (7 items, domain score ranges from 0–28) and work and education (7 items, domain scores range from 0–28). Each item is scored from 0 (very good) to 4 (very poor).

(4) Work-environment and social support

The Dutch and English version of the Job Content Questionnaire (JCQ) [35] was used to measure social and psychological factors at work. The following scales were used: quantitative job demands (5 items, score ranges from 5–20), decision authority (3 items, score ranges from 3–12) and skill discretion (6 items, score ranges from 6–24), and supervisor (4 items, score ranges from 4–16) and coworker support (4 items, score ranges from 4–16). For each item, the response options were: 1 = strongly disagree, 2 = disagree, 3 = agree, 4 = strongly agree. For each scale, we calculated the overall score by summing the response scores of the individual items. Higher scores indicate more job demands, decision authority, skill discretion, supervisor and coworker support. The JCQ is not applicable for students. However, among students social support was measured using the Dutch and English version of the Significant Others Scale (SOS) [36]. The scale contains 12 items scored from 1 ‘no, not at all’ to 5 ‘very clearly’. The total score ranges from 12–60. A higher score indicated more social support.

Physical load during work or study was assessed using the short Dutch and English version of the Dutch Musculoskeletal Questionnaire (DMQ) [37]. The items address force exertions, and static, dynamic and repetitive movements of the upper extremities. Each item is scored on a two-point scale ranging from 1 ‘no’ to 2 ‘yes’. Two separate scores were calculated, namely ‘heavy physical workload ‘(7 items) and ‘long-lasting postures and repetitive work’ (8 items). Higher scores indicate more physical load.

### Statistical analysis

First, characteristics of survey respondents were described for the employee and student population separately. For the purpose of this study, we analyzed data of those employees and students who completed questions on the operationalization of CANS. Chi square tests and independent samples *t* tests were used to test univariate differences between survey respondents who reported to experience CANS versus those who did not, and to examine univariate differences in various types of help seeking behaviour (already sought help yes/ no, intention to seek help yes/no) among employees who were identified with CANS. Separate analyses were performed for employees and students who responded to the survey questionnaire. Differences were described for variables related to the experience of CANS and help seeking behaviour, such as demographic variables, work or study related variables and complaint related factors. A non-response analysis was performed to examine whether respondents who reported CANS, differed in demographics and work or study related variables, compared to those responders who did not report CANS.

Furthermore, non-response analyses were performed to examine whether respondents who gave informed consent for participation in the subpopulation, differed in demographics, complaint characteristics or factors related to help seeking behaviour, compared to those respondents of the survey questionnaire who did not provide informed consent, and to examine whether those who provided informed consent for participation in the subpopulation, but did not respond to the additional questionnaires, differed significantly from those who provided informed consent and did actually fill out the additional questionnaires.

Subsequently, chi square tests and independent samples *t* tests were used to test univariate associations between factors related to functioning and participation, social support, work environment and demographics with help seeking behaviour, in the subpopulation of employees and students with self-reported CANS (selected out of the respondents of the cross-sectional survey).

Statistical analyses were performed using SPSS for Windows, version 21.

## Results

### Descriptive characteristics of survey respondents

Table 1 displays the characteristics of the total population of survey respondents, comprising employees and students of Maastricht University and Zuyd University of applied sciences. A total of 1,768 employees and 3,332 students responded to the survey questionnaire, representing a response rate of respectively 29.6% and 11.9%. The total sample of survey respondents comprised a higher proportion of women. Within the employee sample the majority of respondents have a high educational attainment, work ≥ 3 years in their current job, and have a fulltime employment status.

**Table 1:** Characteristics of survey respondents

	Employees^a^ (*n* = 1765)	Students^b^ (*n* = 3322)
Gender		
Male (%)	36.6	31.2
Female (%)	63.4	68.8
Age mean (SD)	44.7 (11.3)	22.3 (4.2)
Educational level		
Low and medium (%)	20.5	43.0
High (%)	79.5	57.0
Job category		
Support staff (%)	57.3	n.a.
Scientific/teaching staff (%)	42.7	n.a.
Years in current job		
< 3 years (%)	23.4	n.a.
≥ 3 years (%)	76.6	n.a.
Employment/study status		
Fulltime (%)	68.0	95.5
Parttime (%)	32.0	4.5
Current study program		
Bachelor (%)	n.a.	55.0
Master (%)	n.a.	45.0
VDU use work/study related		
< 4 h/day (%)	15.8	44.1
≥ 4 h/day (%)	84.2	55.9

^a^Total of cases does not equal total of survey employees respondents (n=1768) due to missing values

^b^Total of cases does not equal total of survey student respondents (n=3332) due to missing values

*h* hours, *SD* Standard deviation

Figure 1 depicts the flowchart of the survey population. Within the employee population, 639 cases had missing data on one or both of the survey questions on the possible cause of their complaints (i.e. acute trauma or systemic disease) and were therefore excluded from the analysis. Within the student population, 986 cases were incomplete on questions related to the identification of CANS and were excluded from the analysis. This resulted in a total sample of 3,475 cases which were included in the analysis. We compared characteristics of respondents who completed survey questions on identification of CANS, with characteristics of individuals with incomplete responses on questions related to the identification of CANS. No significant differences were observed regarding demographics, work or study related variables.

### Selection of participants of subpopulation

Figure 1 displays the selection process of survey respondents included in the subpopulation. Out of the 239 respondents who provided informed consent and were included in the subpopulation, 189 actually answered additional questionnaires whereas 50 did not answer the invitation to fill out additional questionnaires.

We performed non-response analyses regarding the selection process of the subpopulation out of the respondents of the cross-sectional survey to gain insight in the possibility of self-selection bias. First, characteristics of survey respondents who reported CANS but were not included in the subpopulation (*n* =1157), were compared to characteristics of participants of the subpopulation (*n* = 239). No significant differences were observed regarding demographic characteristics, complaint related variables or factors associated with help seeking behaviour. Second, we compared characteristics of participants of the subpopulation who filled out the additional questionnaires (*n* = 189), with characteristics of those participants of the subpopulation who did not respond to the invitation to fill out the additional questionnaires (*n* = 50). Also, within the latter two populations no significant differences were observed regarding demographics, health related variables or factors associated with help seeking behaviour.

### Experience of CANS and help seeking behaviour

Tables 2 and 3 present the experience of CANS and help seeking behaviour among respectively employees and students.

**Table 2:**
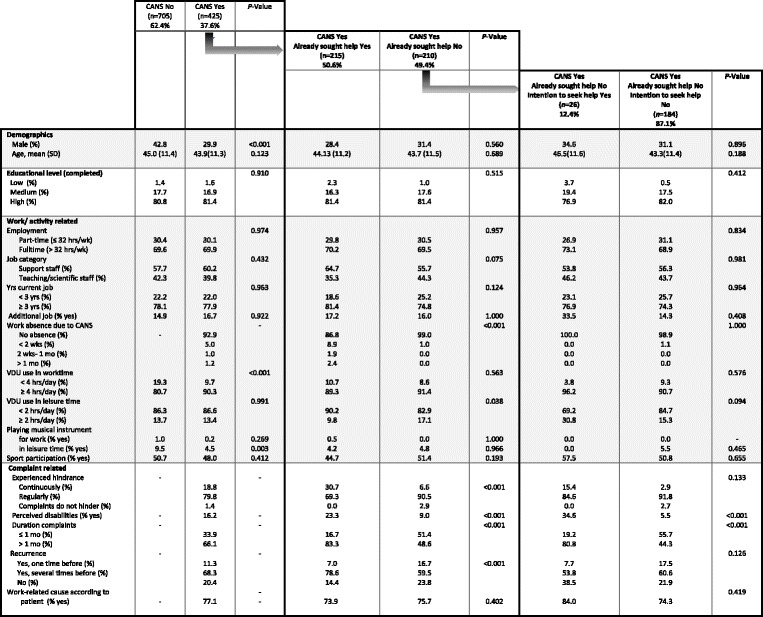
Experience of CANS and help seeking behaviour in respondents of the survey questionnaire –employee population

Legend: *SD* Standard Deviation, *Hrs* hours, *wk* week, *mo* month(s), *VDU* Visual Display Use

**Table 3:**
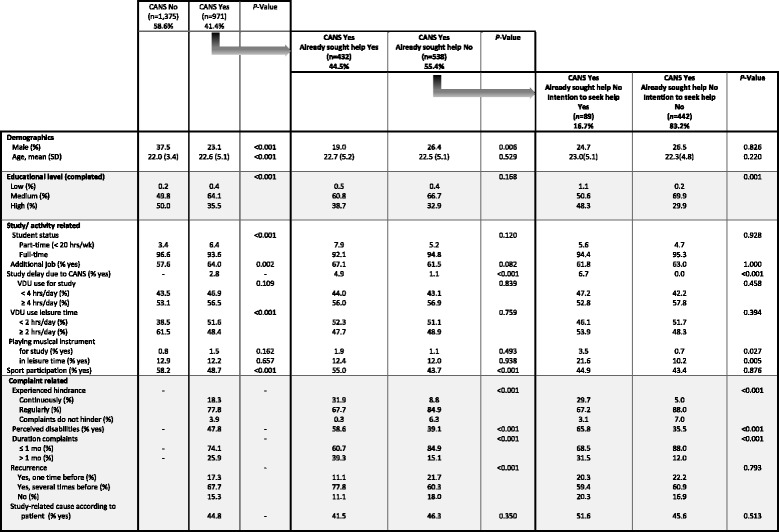
Experience of CANS and help seeking behaviour in respondents of the survey questionnaire – students population

Legend: *SD* Standard Deviation, *Hrs* hours, *wk* week, *mo* month(s), *VDU* Visual Display Use

Table 2 depicts the results of the *employee population*, in which 37.6% reported to experience CANS. With respect to demographics and factors related to work or activities, several significant differences were found between the groups ‘experience of CANS No’ and ‘experience of CANS Yes’. The group entitled ‘experience of CANS Yes’ comprised a significant higher proportion of women and indicated more work related VDU use, compared to the group ‘experience of CANS No’.

As presented in Table 2, of all employees who reported to experience CANS, 50.6% already sought help for their complaints. The majority of employees sought help from more than one health care provider. That is, 98 sought help at a general practitioner, 15 visited an occupational physician, 143 a physiotherapist, 19 visited an exercise therapist and 77 employees received help from someone else. Employees who did not seek help (49.4%) indicated lower experienced hindrance of the complaints, perceived fewer disabilities in daily activities, had shorter duration of complaints, and had less often experienced similar complaints earlier, as compared to those who already sought help for CANS. Of those employees who did experience CANS but did not seek help, 87.1% reported to have *no* intention to seek help for their health complaints in the future. Employees who had not sought help *and* had no intention to seek help perceived fewer disabilities and indicated shorter duration of complaints.

In Table 3, the survey results of the *student population* are presented. Within the student population, 41.4% reported to experience CANS. The students of the group entitled ‘experience of CANS Yes’ included a higher proportion of women, were older, completed a lower educational level, had more often a part-time study status, indicated less VDU use in leisure time and comprised a higher proportion of students whose study involved playing a musical instrument, compared to the group students who reported ‘experience of CANS No’.

Of all students who reported to experience CANS, 44.5% had already sought help for their complaints. Students who had sought help, most often sought help at more than one health care provider. That is, 234 sought help at their general practitioner, 5 at an occupational physician, 297 at a physiotherapist, 44 at an exercise therapist and 140 sought help at someone else (types of health care providers are not displayed in Table 3). Students that had *not* sought help (55.4%) reported, amongst others, to experience less hindrance of their complaints, to perceive fewer disabilities, reported a shorter complaint duration, and less recurrence of complaints, compared to the help-seekers. These differences are not only statistically significant, but also clinically relevant.

Of the students who did *not* already seek help, 83.2% had no intention to seek help for their complaints in the future. Students who had no intention to seek help displayed several statistically significant and clinically relevant differences compared to those who had the intention to seek help. They experienced, amongst others, less hindrance of the complaints and perceived fewer disabilities, and indicated shorter complaint duration.

### Factors associated with help seeking behaviour in the subpopulation

Table 4 displays help seeking behaviour in participants of the subpopulation, and associations with seeking help. Within this subpopulation, 52.7% had sought help for their complaints already, whereas 47.0% had not sought help for CANS. In comparison to participants who had sought help for CANS, non-help seekers reported to experience less frequent hindrance of their complaints, and experienced less discomfort due to CANS. Furthermore, they reported less diagnosed specific CANS, reported less functional limitations (DASH), and less impact on participation and autonomy on all domains of the IPA, except for the domain ‘social life and relationships’. These differences are clinically relevant.

**Table 4:**
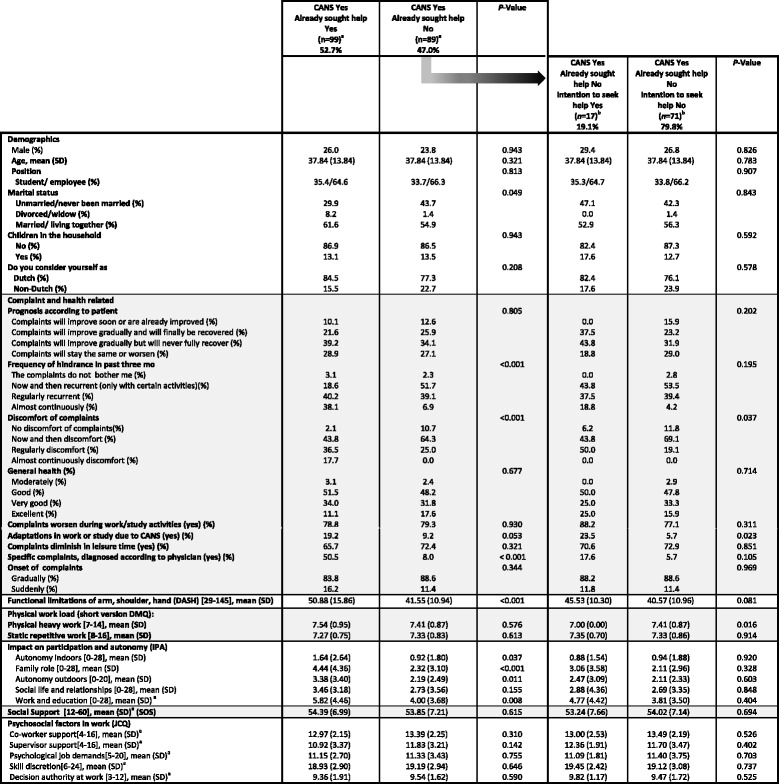
Associations with help seeking behaviour in subpopulation

Legend:[..],score range; *SD* standard deviation, *mo* months, *DASH* Disabilities of Arm, Shoulder and Hand Questionnaire, *DMQ* Dutch Musculoskeletal Questionnaire, *IPA* Impact on Participation and Autonomy questionnaire, *SOS* Significant Others Scale, *JCQ* Job Content Questionnaire

Of the respondents who had not sought help already, 19.1% had the intention to seek help, whereas 79.8% reported to have no intention to seek help for their health complaints. The group that did not seek help yet *and* had no intention to seek help, reported to experience less discomfort due to CANS, made less adaptations in workplace or work tasks, and reported more physically demanding work, compared to respondents who did not seek help yet but had the intention to seek help.

## Discussion

### Main findings

The aim of the present study was to examine whether perceived hindrance is associated with help seeking behaviour in employees and students identified with CANS, and to explore the role of factors related to functioning and participation, work-environment and demographics in help seeking behaviour. The results showed that help seeking behaviour is primarily associated with characteristics related to the perceived hindrance of the complaints and the consequences of these complaints for individual functioning. In addition, the results of the survey questionnaire revealed that a considerable part of the people who reported CANS, did not seek help *and* had no intention to seek help for their complaints either. We found by and large similar results of the survey questionnaire with respect to help seeking behaviour and factors associated with help seeking, in the student population and the working population.

### Methodological and conceptual considerations

To our knowledge, this is the first study investigating help seeking behaviour specifically in a population with self-reported CANS. A strength of this study is that a broad range of variables was included, which are important according to the HBM. The HBM states that health behaviour is primarily determined by the perceived threat of the disease [22–24]. Therefore, we included questions on perceived hindrance, perceived disabilities in daily activities and impact of the complaints on participation and autonomy. Furthermore, we assessed demographic, sociopsychological and complaint related variables which may strongly affect individual’s perceptions and thus indirectly influence health-related behaviour [38].

Our results were to a considerable extent in accordance with the HBM. Factors related to the perceived threat were significantly associated with help seeking behaviour in the majority of analyses. However, it is striking that some factors revealed no significant associations although they are believed to have an indirect effect on health behaviour as stated by this model. For example, no significant associations with help seeking behaviour were observed for personal estimates of prognosis, psychosocial factors in work, and social support. The possibility exists that the absence of associations between these factors and health behaviour is typical for CANS.

A few methodological issues of this study need to be considered. First, the overall response rate for the survey were 29.5% in the employee population, and 11.5% in the student population. These low response rate imply a substantial risk for selection bias. The results of this survey might, therefore, not be a correct reflection of the actual population characteristics. We had no possibility to recontact non-participants and address a nonresponse questionnaire in order to reduce selection bias [39]. Therefore, the effect of potential selection bias in our study remains unclear. Because the survey questionnaire was introduced to the target population as a questionnaire focusing on CANS, it is possible that people who are affected by CANS are more willing to respond than those without CANS. For this reason, an overestimation of self-reported CANS among responders compared to non-responders is possible.

Additionally, a considerable group was excluded from analyses due to missing data on the presence of UEDs, acute trauma or systemic disease. It is reasonable to assume that these data are missing at random (MAR). Complete-case analysis was performed, which implies a certain risk of biased results when data are not completely missing at random (MCAR). However, a non-response analysis revealed no significant differences in demographics, work or study related factors.

Nevertheless, a considerable group which was excluded from analysis indicated to experience UEDs (*n* = 1264) in general but did not indicate whether these complaints could be explained by an acute trauma or systemic disease, and were excluded from analysis. Therefore, it should be noted that prevalence rates of CANS could be underrepresented in this study.

Secondly, the validity of self-reported CANS could be debated. From the literature it is known that much higher prevalence rates of musculoskeletal disorders are found from self-reporting than when estimated from registrations or physical examinations [40, 41]. However, when assessing the presence of a disease that is characterized by pain and functional limitations, the individual is often considered as the single best source of information [40]. According to Picavet et al., the test-retest reliability of self-reported musculoskeletal diseases is fair to good and the consistent correlation with pain make self-reports a useful to measure musculoskeletal health complaints in surveys [40].

### Implications for a preventive strategy for CANS

The results of the cross-sectional survey revealed that approximately 40% of our study population experienced CANS at this moment or within the past three months. However, our definition of CANS allowed inclusion of acute and prevalent cases, and mild as well as severe complaints. One may still wonder, however, whether this total group of people who experience CANS should be defined as target population for a preventive strategy, as we initially suggested. Our results revealed that approximately half of the people who experienced CANS did already seek help or had the intention to seek help. Furthermore, of those who did not seek help *and* had no intention to seek help, a significant proportion did not experience hindrance or had not perceived disabilities due to CANS. These findings might indicate that a considerable part of those experiencing CANS appear to be able to make a sound self-assessment of the severity of their complaints and the necessity to seek help. However, on the other hand, the group non-help seekers still comprised a substantial proportion of employees and students who did experience hindrance or had perceived disabilities due to CANS. Despite the perceived severity of their complaints, they appear to have no intention to seek help and are unlikely to participate in a preventive strategy. It is expected that particularly these people would greatly benefit of a preventive strategy directed towards them. Consequently, the results of our study encourage a reconsideration of the definition of the target population for an early preventive strategy. Such a definition should include the presence of CANS on the one hand, and a quantification of the severity of the complaints, such as the level of perceived hindrance or disabilities, on the other hand. In line with this definition, the selection process should be two-fold in order to detect this target population. This implies a validated screening instrument with not only the ability to detect the presence of CANS, but also the possibility to assess the severity of the complaints and help seeking behaviour in relation to the complaint. In the process of development of a valid screening instrument*,* an optimal cut-off point has to be chosen, based on ideally both high specificity and sensitivity, as well as on predictive values. Although an early preventive strategy for CANS is not harmful when applied to the wrong person (false positives), people are wrongfully labelled as unhealthy. Moreover, applying a preventive strategy to the wrong person may lead to unnecessary costs. To ensure that preventive strategies are directed at those who will benefit the most, choices should be made towards a screening instrument with high specificity and a positive predictive value, in order to reduce the number of false positives.

To enhance participation of a target population in a preventive strategy, screening uptake must be optimal. However, when the target population receives a screening invitation for CANS, it is expected that they are unlikely to respond as intentions to seek help appear limited. Moreover, the relative low response rate in our study reflects the possible dilemma of low screening uptake. To optimize screening uptake, the screening invitation should be customized to the specific characteristics of these low-uptake groups and should, e.g., address knowledge, risk perceptions, awareness and misconceptions. The results of this study could contribute to a tailored screening invitation. When applied to the target population in our study, a tailored screening invitation might include explicit information on the importance of timely identification and early interventions, and the urgency to participate particularly when complaints do hinder in daily activities or disabilities in functioning are perceived. Furthermore, screening invitations could be customized to educational attainment, job position or hours of VDU use.

As such, the findings of this study provide direction towards the characteristics and selection of the target population and offers guidance in the development of tailored preventive strategies.

### Recommendations for future research

In addition to the experienced hindrance, there are numerous other factors that influence participation in a prevention program, such as the fear that the health complaints might become known to supervisor or colleagues, leading to job loss, or the possibility to participate during work time. According to the HBM, also factors such as personality, knowledge, religion or culture, and individual’s personal considerations as perceived benefits or perceived barriers for help seeking behavior, influence the decision to participate. However, we did or could not include these factors in the present study. For upcoming studies, it would be valuable to examine the associations of these factors with help seeking behaviour within a population of CANS, in order to further customize screenings strategies. Furthermore, future studies might examine whether or not a self-reported ‘intention to seek help’, does result in actual help seeking in the future and to examine which factors have an influence on this process, in order to optimize participation in a preventive strategy. It is also valuable for future research to study different time windows in terms of duration or recurrence of CANS, for the determinants of help seeking behaviour.

## Conclusions

To conclude, as earlier studies have demonstrated that upper-extremity complaints [5] are a major cause of sickness absence and an early intervention might be effective in preventing disabling complaints [15], this study adds that a substantial part of people with CANS display no help seeking behaviour which might be a bottleneck in the implementation of preventive strategies. Individuals who had not sought help *and* had no intention to seek help experienced less hindrance in daily activities, perceived fewer disabilities in functioning, had a shorter duration of complaints and experienced less impact of their complaints on participation and autonomy. Notwithstanding this, a proportion of this latter group did experience hindrance and perceived disabilities but did not display any help seeking behaviour. Precisely these people are expected to be at risk for future sickness absence and/ or severe health complaints, and might greatly benefit from a preventive strategy in an early stage, before severe (work) disability and absenteeism actually occur. Future preventive strategies for CANS, should be tailored to these determinants of help seeking behaviour, in order to optimize screening uptake and participation.

## Abbreviations

CANS: Complaints of Arm, Neck and/or Shoulder
DASH: Disabilities of Arm, Shoulder and Hand questionnaire
DMQ: Dutch Musculoskeletal Questionnaire
HBM: Health Belief Model
IPA: Impact on Participation and Autonomy questionnaire
JCQ: Job Content Questionnaire
SOS: Significant Others Scale
UEDs: Upper Extremity Disorders
VDU: Visual Display Unit
